# The Adoption Potential of Extended Lactation as a Strategy to Reduce Excess Calf Numbers in Dairy Farming

**DOI:** 10.3390/ani14213115

**Published:** 2024-10-29

**Authors:** Josephine Gresham, Christoph Reiber, Mizeck G. G. Chagunda

**Affiliations:** 1 Department of Animal Breeding and Husbandry in the Tropics and Subtropics, Institute of Agricultural Sciences in the Tropics (Hans-Ruthenberg Institute), University of Hohenheim, 70599 Stuttgart, Germany; 2 Demeter Baden-Württemberg, 70771 Leinfelden-Echterdingen, Germany; 3 Centre for Tropical Livestock Genetics and Health, University of Edinburgh, Easter Bush Campus, Midlothian EH25 9RG, Scotland, UK

**Keywords:** dairy cow, dairy calf, organic dairy production, extended lactation, calving interval, milk production, surplus dairy calves

## Abstract

Dairy production relies predominantly on a few, selectively bred, high-yielding dairy breeds, resulting in a surplus of low-value male offspring. This situation leads to a conflict between a growing demand for milk and low appreciation for dairy calves. Extending lactation could reduce the number of calves born. This study aimed to assess the current use of extended lactation in dairy production in Southern Germany, identify the perceived potential, and determine the biological potential extended lactation has to reduce calf numbers. A survey from 2020 with 310 farmers was analysed. A total of 145 (46.8%) farmers used extended lactation. The use of extended lactation on farms correlated significantly with breed, milk yield, interest in continuing or implementing the use of extended lactation, and the indicated feasibility of extended lactation. The perceived potential was assessed by 39.8% of farmers as “high” to “very high”. A total of 17 (12.8%) farmers currently not using extended lactation were identified as potential adopters. Together with existing adopters, this could result in 52.3% of farms using extended lactation, which could biologically reduce calf numbers by 7.3% or 14.1% p.a., when extended by three or six months, respectively. This adoption potential shows that extended lactation can reduce calf numbers considerably, addressing both ethical and economic concerns in dairy farming and benefiting farmers as well as society.

## Simple Summary

Dairy farming relies heavily on high-yielding breeds, but this focus on milk production often leads to low-value male offspring. This creates a conflict: while milk demand rises, the appreciation for dairy calves remains low. Extending lactation periods in cows could help address this issue by reducing the number of calves born. Our study in Southern Germany analysed current extended lactation use, farmers’ interest in it and feasibility, adoption potential, and its biological impact on calf numbers. We surveyed 310 farmers and found that 46.8% used extended lactation. In total, 12.8% of surveyed farmers were identified as potential adopters. If adopted by more farmers, this could reduce calf numbers significantly, benefiting both the farming community and society by addressing economic and ethical concerns in dairy production.

## 1. Introduction

Modern dairy production relies predominantly on a few, selectively bred, high-yielding dairy breeds. This is the case in both conventional and organic systems. Since milk production is a trait expressed only by females, males, even though they carry genes that are important for milk production, do not produce milk, resulting in them having little to no economic value [1]. Since a dairy cow can only produce milk after calving, it is inevitable that calves are produced if the goal is to produce milk. Male and female calves not needed for breeding or replacement in the dairy sector require an alternative use. However, the lower fattening abilities of dairy breed calves compared to beef breeds make them unpopular for fatteners and less profitable for beef production [2]. This situation leads to an imbalance between the growing demand for milk and the number of produced and required dairy calves to meet the demand for veal and beef, as this is met by the supply from beef breed herds [3].

This situation is particularly amplified in the organic dairy sector, even though many organic dairy producers rely on dual-purpose breeds [4,5]. The organic regulations of the European Union (EU) state that organic calves must be fed organic whole milk for the first 90 days of life [6], which increases the rearing costs for the dairy farmer and decreases the profitability and competitiveness of organic calves compared to their conventional counterparts, beef breeds and culled organic dairy cows on the market [2,7]. Subsequently, the few organic fatteners present in Germany fatten mostly beef breeds from suckler cow herds [8], and many of them are not located in Southern Germany, where organic dairy farming is predominantly located. The majority of organic dairy calves are sold to conventional veal or beef producers at a few weeks of age, often younger than conventional calves to minimize the high feeding cost [9,10]. Assessing 287,289 calf sales between 2014 and 2018 in the state of Baden-Württemberg in Germany, Reiber et al. [10] found that on average organic calves were sold at €3.45 less compared to calves from conventional farms. Further, all calves were sold solely into conventional systems, if sold through official calf trading markets. The move of organic calves into the conventional fattening system is not in line with the principles of organic livestock farming [1,11]. Since conventional veal and beef fattening farms are frequently located in regions beyond Baden-Württemberg, in other countries, or outside the EU [10], the result is often stressful and long journeys for the young calves [12]. Public concern about the so-called “calf issue” in organic and conventional systems is rising steadily, and demands for solution strategies are urgently needed [4,9].

A common solution to this issue is to improve the value of the calves by cross-breeding with beef breeds. This strategy is often combined with the use of sexed semen, where the top-performing dairy cows in the herd are inseminated with sexed semen to produce female calves intended as replacement heifers [13]. The rest of the herd is cross-bred with beef breeds to produce calves with desirable fattening traits. However, the use of sexed semen is not permitted for dairy farms with Demeter certification [14].

One alternative solution to the calf issue is the reduction of the number of calves born within a cow’s lifetime. Most dairy cows are managed on a 305-day lactation, typically leading to the production of one calf per cow per year [15]. The lactation period can be extended by deliberately delaying the next breeding cycle, continuing milking, delaying insemination, and consequently extending the calving interval (CI) [16]. Studies from Australia [17,18], New Zealand [19,20], and Ireland [21] have shown that cows are capable of extended lactation up to 670 days, corresponding to a 24-month calving interval. It is a well-known strategy in organic dairy goat production, where it is used to balance the demand for goat milk with the difficulty of marketing kid meat [22]. Similar to its effect in dairy goats, extended lactation and CI in dairy cattle leads to fewer calvings per cow per year and fewer dry days per cow per year, when calculated over the productive lifetime, and fewer replacement heifers [23]. As calving poses an increased health risk, extending CI decreases the health and welfare risks associated with calving, such as lameness, mastitis, and metabolic issues in early lactation, and can improve the longevity of dairy cows [24]. The voluntary delay in rebreeding avoids insemination at the time of peak milk production and may allow for easier conception as the cow is in a stage of more positive energy balance, which can improve pregnancy rates [25]. Dairy cows managed with extended lactation and extended CI can either maintain or slightly increase their daily milk yield and can increase lifetime milk production with either unchanged or improved quality properties in the produced milk [23]. Further, extended lactation can improve the sustainability of dairy production by lowering land use and emissions of greenhouse gases [26]. Farm economics can be improved through extended lactation but depend on the ability of the farm management to realise saved costs and to utilise freed feed-growing land to grow other profitable crops as well as freed barn space for more cows [26]. However, to prolong lactation the persistency of the lactation must be increased, which is influenced by genetics, parity, pregnancy stage [23], nutrition, and milking frequency [27]. The ideal combination of factors to support extended lactation and tools to predict which cows are most suited for extended lactation are the subject of further studies. Although there has been increased discussion on the potential of extending CI to reduce the number of excess calves born in conventional dairy systems, few studies have examined extended lactation in organic dairy farms. Where such studies are available, they have demonstrated that there might be a possibility to half the number of animals being born and slaughtered, while producing the same amount of milk [28].

Apart from studies on the biological potential of applying extended lactation as a strategy to reduce excess calves, knowledge of farmers’ perspectives on the potential of such a strategy and the likelihood of adoption in organic dairy farming is scarce. The current study, therefore, aimed at (1) identifying the current use of extended lactation in dairy production in Southern Germany; (2) identifying the perceived potential of extended lactation; and (3) determining the biological potential extended lactation has to reduce calf numbers.

## 2. Materials and Methods

Data were obtained from a farmer survey conducted within a project which examined the current use and adoption potential of various breeding, husbandry, and marketing strategies to combat the issue of surplus calves in the state of Baden-Württemberg. The survey was programmed in QuestBack Unipark (EFS Fall 2020) and disseminated in an online and print version in September 2020 by the Rinderunion Baden-Württemberg e.V. (Herbertingen, Germany) to their approximately 3500 member dairy farms. At the same time the organic certifiers Bioland, Demeter, and Naturland sent out the survey to their member farms, and the organic trader rebio (Rottenburg, Germany) e-mailed the link to 300 of their supplying farmers. Through the organic certifiers, the survey also reached farmers in Bavaria. The Badische Bauernzeitung also advertised the survey in their monthly publication at the time. The survey was concluded on the 31 October 2020.

The survey included open and closed questions concerning the farm, the dairy herd, and the farmer’s personal information, as well as their current breeding, husbandry, management, and marketing strategies used. Further, farmers were asked to express their interest in the ten different solution strategies presented in the survey in the areas of breeding, husbandry, and marketing, and the feasibility of each strategy, using a 6-point Likert-type scale [29]. In order to avoid a mid-point typically indicating “neither agree nor disagree” as often occurs in 5-point Likert scales, a 6-point Likert-type scale was constructed. This decision ensured that farmers always expressed a tendency of their interest or their assessment of feasibility. The scale for feasibility was defined with 1 as “very feasible” and 6 as “not feasible at all”. The numbers between 1 and 6 were not specifically defined. The farmers were also asked to indicate their interest in continuing the use of or implementing the given strategy on their own farm. Again, they had to rate their answer on a scale from 1 to 6. Here the number 1 was defined as “very interested” and 6 as “not interested at all”. In addition, farmers had the opportunity to add comments to their assessment of interest and feasibility of each strategy.

To identify the current use of extended lactation and to describe the farms using it, a descriptive analysis of the farms, their use of extended lactation and correlating factors was conducted using IBM SPSS version 26. For inferential statistics, the selected variables were checked for normal distribution. Since the variables were not normally distributed, the phi/Cramér’s V test was used to show the statistical relationship between nominally scaled variables. In order to analyse the herds according to their breed, it was decided that herds comprising at least 75% of one breed would be classified as a herd of the according breed (e.g., a herd with >75% HF was considered an HF herd). Herds with no dominating breed (<75%) were categorised as mixed herds.

The perceived potential was measured by the combination of the farmer’s interest in using or continuing the use of extended lactation with their assessment of its feasibility. Their answers were collected using a 6-point Likert-type scale for both interest and feasibility, as described earlier, and additional qualitative data were collected in a comment section. All farmers not yet using extended lactation and expressing “high” to “very high” potential were identified as potential adopters. These results were used in combination with the biological potential to analyse the effects extended lactation could have on calf numbers if the perceived potential was fully converted to adoption by farmers not yet using extended lactation.

While the quantitative data from the Likert scales were analysed using IBM SPSS version 26, the qualitative answers of the farmers were coded in MAXQDA 2020 according to the qualitative content analysis of Mayring [30]. Theoretically each qualitative answer of a farmer could have more than one code assigned to it. Therefore, there were more codes assigned than qualitative answers given. The list of codes and categories was prior established with others who were analysing the other strategies included in the survey, contributing to further studies. This would enable a later consolidation of all results of the different studies for project purposes. A total of 171 qualitative answers were collected from the 310 completed surveys. Pre-defined codes were sorted within categories of “animal performance”, “operational management”, “animal health”, “implementation”, “demand”, “(personal) rejection/acceptance”, “ethics/values/animal welfare”, “efficiency of the technology/strategy”, “business economics”, “undefined”, and “marketing/market”. Overall, 309 codes from all eleven categories were assigned to the 171 qualitative answers through qualitative content analysis.

Four scenarios were chosen to calculate the biological potential extended lactation would have on reducing calf numbers. The scenarios are based on performance data from 2020 collected by Landesverband Baden-Württemberg für Leistungs- und Qualitätsprüfungen in der Tierzucht e.V. (LKV) (Stuttgart, Germany) [31]. According to the milk yield recording (MLP) conducted by the LKV in the state of Baden-Württemberg, the mean age of dairy cows at their end of use was 6.6 years, at which time they had completed a mean 4.1 lactations with a mean CI of 416 days. To calculate different scenarios of extended lactation periods, the 416-day CI was used as the standard CI, as the farmers already using extended lactation in the survey did not indicate the length of extension and the 416-day CI exceeds the standard 395-day CI (on the basis of a 305-day lactation) by only 21 days.

Using the mean number of lactations of a dairy cow in Baden-Württemberg of 4.1 as a fixed variable and the mean standard lactation length in Baden-Württemberg of 416 days, the length of those lactations was extended by three months (scenario 1) and by six months (scenario 2). The third and fourth scenarios calculated the effect of extending lactations by three and six months, respectively, without extending the productive life of the dairy cow, resulting in fewer mean lactations per cow. Scenarios 1 and 3 therefore tested lactations with an overall length of 506 days, and scenarios 2 and 4 tested 596-day lactations.

To calculate the effects of the adoption potential on the number of calves born annually, the current adopters and potential adopters were combined and subjected to the four scenarios. It was assumed that cows were distributed evenly on all farms, resulting in 52.3% of all dairy cows in Baden-Württemberg performing an extended lactation of three or six months in the four scenarios.

## 3. Results

### 3.1. Description of Respondents and Farms

The survey was accessed 1024 times and completed by 310 dairy farmers (response rate 30.3%), of which 209 were organic dairy farmers. Although dairy farmers in southern Germany (states of Baden-Württemberg and Bavaria) were specifically targeted, only 84.5% of the organic farmers who participated were from these two states. Of the total 310 farmers, 13% did not give information pertaining to their location. Of the organic farmers, 2.4% were from other German states (Brandenburg, Hessen, Niedersachsen, Rheinland-Pfalz), due to the irregular dissemination through some organic certifiers outside the target region.

Most organic farms that participated were members of an organic farmers association, certified by Naturland (40.7%) organic association, followed by Demeter (29.2%) and Bioland (26.8%). More than twice as many organic farms (45.0%) belonged to a farmer cooperative than did conventional farms (20.8%).

Table 1 shows the number of cows and the breeds on organic and conventional farms. On average, conventional farms had considerably larger herd sizes (76 cows) compared to organic farms (43 cows). In total, the mean number of cows per farm was 54.3. The share of Fleckvieh cows was higher on organic farms (52%) than on conventional (43%), while the Holstein Friesian was higher on conventional (46%) than on organic farms (24%).

Of the 310 farms, a total of 81.0% participated in milk performance testing (MLP). For the conventional farms, the share of farms participating in MLP was higher (94.1%) than that of organic farms (74.6%). The mean amount of milk produced per cow per annum was 7048.68 litres (L), and the mean annual milk production per farm was 431,765.28 l. The mean number of female calves kept on the farms as replacement heifers was 19.38 (36.26%).

### 3.2. Current Use of Extended Lactation and Farms Implementing It

As shown in Table 2, about half of the 209 organic dairy farmers indicated having implemented extended lactation at the time of the study (102, 48.8%), while slightly fewer conventional farmers indicated that they used extended lactation (43, 42.6%).

Correlation tests, seen in Table 3, indicated a highly significant correlation (*p* < 0.001) between the breed and implementation of extended lactation. Further significant positive correlations were found between the current use of extended lactation and milk production per cow (*p* = 0.032), interest in continuing the use or introducing the use of extended lactation (*p* < 0.001), and the feasibility of extended lactation (*p* < 0.001). The farm type (organic or conventional) and the participation in milk yield recording (MLP) had no significant correlation.

As shown in Table 4, herds with more than 75% Holstein Friesian cows had the highest use of extended lactation with 68.3%, followed by mixed-breed herds with 64.0% (herds with less than 75% of cows of one breed) and Braunvieh herds with 57.6%. Of the Fleckvieh herds, only 37.4% used extended lactation. Vorderwälder and other breed type herds had an even lower use of extended lactation at 25.9% and 22.2%, respectively.

### 3.3. Perceived Potential of Extended Lactation and Relationship Between Perceived Potential and Biological Potential

#### 3.3.1. Expression of Interest and Feasibility

Of the 310 dairy farmers, 195 (93.3%) of the organic farmers and 89 (88.1%) of the conventional farmers indicated how feasible they consider extended lactation, while 186 (89.0%) of the organic farmers and 88 (87.1%) of the conventional gave indications of their interest in continuing the use or introducing extended lactation.

As Table 5 shows, the mean feasibility was 3.08 and mean interest was 3.26 for all farmers, both lying close to the median of the Likert scale (1–6), indicating “moderate” scores for both feasibility and interest in the strategy. When organic farmers were considered separately, the mean had a minimal change to 3.09 and 3.19 for feasibility and interest, respectively.

The correlation test, see Table 6, indicated a significant negative correlation between the expressed interest in continuing the use or introducing extended lactation and the amount of milk produced per cow p.a. The same factor also significantly correlated with the expressed feasibility. This negative correlation signifies the lower the milk production by the cows, the higher the interest in extended lactation and the higher the perceived feasibility by the farmer.

#### 3.3.2. Perceived Potential of Extended Lactation

To obtain the perceived potential of the strategy of extended lactation the expressed interest and feasibility were combined. A mean score between 1 and 2.5 given for interest and feasibility was considered as having a “high” to “very high” perceived potential. Mean scores between 3 and 3.5 were categorised as “moderate” perceived potential, scores of 4 to 5.5 as “low” to “very low” perceived potential, and mean scores of 6 were considered as “no” perceived potential.

To identify potential adopters, only farmers currently not using extended lactation were considered. As Table 7 shows, 55 (41.4%) farmers currently not using extended lactation showed a “low” to “very low” perceived potential of the strategy, while 17 (12.8%) farmers currently not using extended lactation indicated a “high” to “very high” perceived potential.

#### 3.3.3. Adoption Potential

To assess adoption potential, farmers not yet using extended lactation and expressing “high” to “very high” potential had to be identified, as these are the potential adopters. A share of 12.8% of all farmers were identified as potential adopters, which would increase the use of extended lactation from 46.8% to 52.3%. Within the organic sector alone, an increase from 48.8% to 55.5% could be achieved potentially.

#### 3.3.4. Qualitative Answers of Farmers Regarding Their Expressed Interest and Feasibility

Following the questions regarding interest and feasibility, farmers were asked to comment on their answers, explaining or reasoning their answers on the Likert scale. A total of 171 qualitative answers were collected from the 310 completed surveys. Pre-defined codes were sorted within categories of “animal performance”, “operational management”, “animal health”, “implementation”, “demand”, “(personal) rejection/acceptance”, “ethics/values/animal welfare”, “efficiency of the technology/strategy”, “business economics”, “undefined”, and “marketing/market”. Overall, 309 codes from all eleven categories were assigned to the 171 qualitative answers. The three codes with the highest occurrence were “reduced milk performance” with 28 hits (9.1%), belonging to the category “animal performance”, “not suited/required, because of dual-purpose breed” with 20 hits (6,5%), and “good persistency required” also with 20 hits (6.5%). These top three codes can all be connected to the biology of the cow.

### 3.4. Biological Potential of Extended Lactation to Reduce Calf Numbers

Table 8 shows the four different scenarios of extended lactation that were calculated. Lactation was extended by three and six months, once also extending the productive life of the cow and once without extending the productive life of the cow. For scenarios 1 and 2, the extension of the productive life resulted in an extension of lifespan to 7.61 years and 8.62 years, respectively. It did not change the number of calves born in the cow’s lifetime but reduced the number of calves born per cow from 0.84 calves/annum to 0.72 calves/annum in scenario 1 and to 0.61 calves/annum in scenario 2 during their productive life.

In scenarios 3 and 4, similar results were observed. However, as the productive life of the cows was not extended in these scenarios, the number of calvings per cow lifetime was reduced, as was the number of lactations. In scenario 3, the cows would only perform 3.37 lactations and produce 0.51 calves/annum in their lifetime. The lowest amount of calvings per cow was observed in scenario 4, with cows having 2.86 lactations and 0.43 calves per year during the lifetime (see Table 8).

As identified in 3.3.3., the annual number of calvings in Baden-Württemberg could be reduced by 7.34% with the application of scenarios 1 or 3, and by 14.14% when applying scenarios 2 or 4.

## 4. Discussion

Foremost, the results show, at the time of the questionnaire, a notable use of extended lactation as a management strategy by farmers in Southern Germany, surpassing expectations based on previous records from LKV on CI [31]. Further, the results highlight that extended lactation is perceived as having moderate to very high potential for the farmers interviewed. Although the perceived potential of extended lactation has various explanations as shown by the qualitative results, the leading codes all related to either the biological potential or limitations of extended lactation. Another key finding was that utilising the biological potential of extended lactation of dairy cows in the state of Baden-Württemberg could reduce the number of calves born per annum, which can help alleviate the calf issue.

The current use of extended lactation identified was higher than expected, not just based on the records of LKV, but also based on the fact that common recommendations provided to German farmers recommend CI of less than 400 days [31,32]. However, some of these recommendations date back to agricultural education of the 1970s, which relied on data from cows producing around 3500 kg of milk per lactation, less than half the yield of most cows today [33]. These reference levels are long outdated, yet seem to still influence many management decisions on farm. The observed results suggest that there may be more farmers who have adapted their CI management to suit the performance levels and biology of modern dairy cows. As shown by Römer et al. [33], CI has been gradually increasing as milk performance and persistency in dairy herds have increased, and fertility rates have decreased.

Furthermore, rising awareness about the calf issue and familiarity with mitigation strategies may have influenced the acceptance of alternative management strategies, such as extended lactation, positively. Farmers who are more aware of the calf issue and familiar with the potential mitigation strategies were more likely to participate in the questionnaire. Consequently, the actual overall use of extended lactation may be lower than indicated.

The results reinforced the suitability of extended lactation and CI for high milk-yielding breeds, on the one hand, through a higher usage rate in these breeds such as the Holstein Friesian. As previously stated by Sehested et al. [23], these breeds often possess the required persistency, showing the highest use of extended lactation among all breeds. Breeds with lower milk yields, such as the Vorderwälder, showed very low use of extended lactation, supporting the assumption stated before, and aligning with the correlation found between the use of extended lactation and the cow’s annual milk yield.

Contradictory to these findings, the interest and feasibility in continuing the use or introducing the use of extended lactation expressed by the farmers correlated negatively with the farm’s annual milk yield per cow. This result contradicts the positive correlation found between current use of extended lactation and higher milk/cow/annum. The literature supports the latter, as high milk-yielding breeds with good persistency have been found to be better suited to extended lactation [23]. One explanation for the negative correlation could be that even though current non-users, the potential adopters, within this group have low milk/cow/annum performance at that moment, they do recognise the potential of extended lactation for their farm or for dairy production generally.

The statements on the suitability of extended lactation provided by the farmers coincided with the lower use of extended lactation in dual-purpose breeds (Vorderwälder and Fleckvieh herds). It is only when the biological potential, such as high persistency, and the will for adoption are available that extended lactation can be successfully implemented. These dual-purpose breeds are at times found on farms with seasonal calving, which links peak lactation to the seasonality of the feed supply, which would not allow for extended lactations. However, in Germany, seasonal calving is very uncommon [34]. Nevertheless, extensive and low input as well as some organic dairy systems, where commonly dual-purpose breeds are kept, depend on seasonally available forage and may struggle to maintain sufficient milk production to extend lactations.

Although the results concerning perceived potential showed that only 39.8% of questioned farmers identified “high” or “very high” potential in extended lactation, 46.8% of the farmers indicated that they used extended lactation at the time of the survey. This may suggest that even those farmers not expressing “high” or “very high” potential perceive extended lactation as useful and utilise it. An Australian study from 2004 looking at dairy farmer interest in extended lactation found that only 10% of respondents had implemented extended lactation [35]. However, the authors identified more potential adopters, with 30% of their survey respondents perceiving extended lactation as a suitable strategy for their dairy herds. Contrary to the present study, the farmers in the 2004 study expressed the perceived benefits of extended lactation to include greater and more consistent production, whereas the most frequently mentioned disadvantage by the farmers in this study was “reduced milk performance”. Yet, the results from both studies suggest that the perceived potential of extended lactation is highly dependable on the level of persistency of milk production. Interestingly, a further contradiction was found between the study by O’Brien and Cole [35] and the present study. They found that farmers perceived improved conception rates as a further benefit of extending lactation. This may result from the delay of insemination to a time after peak lactation, when the energy balance of cows has turned positive. This was not perceived by farmers in this study. One explanation could be that the surveyed farmers may have had fewer issues with fertility in their herds due to the relatively high use of dual-purpose breeds compared to the herds studied by O’Brien and Cole. Another explanation could be that some farmers harbour concerns about reduced fertility in cases of delayed insemination [36]. The most apparent explanation is that the surveyed farmers were not focused on fertility issues but rather on strategies to mitigate the calf issue, which may explain why other benefits of extended lactation, such as its effects on fertility, may not have been recognised.

The results from the scenario calculations indicate that combining the biological potential with the current and potential adopters can reduce the number of calves born annually. Both scenarios 1 and 2 do not change the number of calves born per lifetime of a cow, however the number of required replacement heifers is consequently lower, as the lifespan of the cows was extended [23]. This in turn could diminish the effect extended lactation can have on reducing calf numbers, as more heifers would not be needed as replacements. It can also result in longer generational intervals and consequently effect a genetic lag [16]

Scenarios 3 and 4 also show the same reduction of calves born per year during the productive years of a cow; however, the number of calves over the lifetime is also reduced, as the lifespan is not extended. The cows perform fewer lactations in these scenarios, consequently producing fewer calves in their lifetime. The replacement rate of the herd is unchanged, although the number of calves born that are required as replacement heifers increases. These scenarios were calculated even though extended lactation and CI is expected to also improve the longevity of cows [24]. Nevertheless, there are other factors, such as, for example, illness and lameness, that may affect the lifespan.

All four scenarios theoretically reduce the number of calves born; nonetheless, considering the qualitative responses from the farmers, it is best that the suitability of the individual cow and herd is considered when planning to extend lactation. Scenarios 1 and 2 might be ideal strategies for high milk-yielding breeds or cows with proven persistency in milk performance to reduce calf numbers, while achieving health benefits and increasing longevity.

To ensure sufficient and best-suited replacement heifers are produced to guarantee the future of the dairy herd, the use of sexed dairy semen can be combined with extended lactation and CI [16]. This could, for one, compensate for the genetic lag caused by extended lactation, and, secondly, allow the use of beef semen to produce cross-bred calves for beef production. This in turn can reduce the issue of surplus calves even further than the strategy of extended lactation alone.

The results further confirm the potential recognised by other authors in extending lactation and CI to reduce the number of calves being born in dairy farming [1,26,28,36,37]; however, they do so on the basis of a different methodology, a survey, utilising farmers’ perspectives and experiences. The described potential is, however, not limited to the perceived or biological potential, but also extends to the ethical and environmental consequences of producing fewer calves. Fewer calves and longer productive lives of cows result in fewer replacement heifers being raised, which can reduce the environmental impact of dairy farming and lower greenhouse gas emissions [15,38,39]. Fewer calves also lead to fewer animals being slaughtered, which may reduce consumers’ concerns connected with animal slaughter, especially the slaughter of young animals [28]. The possible improvement of cow health and longevity through extended lactation and CI may also appease ethical debates surrounding intensive dairy production, which puts great strain on dairy cows [1]. Overall, the ethical implications of extending lactation and CI is an area that is in need of further research.

The motivation of farmers to extend lactation and CI may not exclusively be to reduce surplus calves, but may also include economic, animal health and welfare, managerial, or ecological reasons. Some farmers may choose to extend lactation and CI to increase milk yields, improve consistency of income, to increase the time between critical transition events such as calving and drying off, or to reduce farm labour [35,40]. Farmers may also want to deliberately reduce dry days per year and delay pregnancy effects on milk yields. Reasons for unintended extended lactation and CI, such as fertility issues or management issues surrounding heat detection, could also have benefited from further analysis; however, this will have to be subject of future studies.

Calculating the change in replacement rate was not in the scope of this study, but it is a clear limitation to the calculation of the potential extended lactation has to reduce calf numbers. Future calculations should consider potential changes to the replacement rate due to extended lactation. Another limitation of the study is the lack of information regarding the duration of the applied extended lactation and whether the extension was deliberate or unintended. The questionnaire did not provide a definition of the term “extended lactation”, allowing for considerable room for interpretation.

## 5. Conclusions

Based on the findings, extended lactation holds considerable potential as a strategy to mitigate surplus calf numbers in Southern German dairy farming. This study reveals a higher adoption rate than anticipated, driven by factors such as breed suitability and milk yield, but also by farmers’ perceptions of its potential. This considerable potential must be utilised to inform, support, and encourage farmers to use extended lactation not only to reduce the calf issue but to take advantage of the other benefits it can bring. Biologically, extending lactation periods could significantly reduce annual calf numbers, additionally offering potential environmental and economic benefits, and addressing ethical concerns. However, future research should explore factors like replacement rates and unintended lactation extensions. In summary, extended lactation presents a multifaceted approach to not only reducing the number of surplus calves, but to enhancing dairy farming sustainability and efficiency.

## Figures and Tables

**Table 1:** Cow numbers and breeds used in organic and conventional dairy farms.

		Bioland (*n* = 52)	Demeter (*n* = 59)	Naturland (*n* = 84)	Not Known (*n* = 4)	Total Organic (*n* = 199)	Conventional (*n* = 94)	Total (*n* = 293)
Fleckvieh	Mean	20.77	21.73	24.08	13.25	22.30	32.73	25.62
	%	40.18	56.48	58.10	62.35	52.05	42.98	47.94
Holstein Friesian	Mean	19.10	7.36	7.43	1.50	10.34	35.26	18.27
	%	36.94	19.12	17.92	7.06	24.14	46.30	34.19
Braunvieh	Mean	7.13	5.95	6.99	0.75	6.59	5.97	6.39
	%	13.80	15.46	16.86	3.53	15.38	7.84	11.96
Vorder-wälder	Mean	2.15	0.00	0.00	3.75	0.64	0.99	0.75
	%	4.17	0.00	0.00	17.65	1.49	1.30	1.40
Other breeds	Mean	2.54	3.44	2.95	2.00	2.97	1.20	2.41
	%	4.91	8.94	7.12	9.41	6.93	1.58	4.50
Total number of cows	Mean	51.69	38.47	41.45	21.25	42.84	76.15	53.45
	%	100.00	100.00	100.00	100.00	100.00	100.00	100.00

**Table 2:** Current use of extended lactation.

	Organic Farms (*n* = 209)	Percentage (%)	Conventional Farms (*n* = 101)	Percentage (%)
Yes—extended lactation is used	102	48.8	43	42.6
No—extended lactation is not used	107	51.2	58	57.4

**Table 3:** Correlation test using a chi-squared test of farms with extended lactation and other factors.

	Pearson Chi^2^	*p*	*n*
Farm type (organic or conv.)	1.061	0.303	310
Herd type (breed)	25.832	>0.001 **	304
Milk yield recording (MLP)	0.476	0.490	304
Number of cows in herd	−0.062	0.290	294
Milk (L)/cow/annum	0.127	0.032 *	286
Milk production (L)/farm/annum	−0.017	0.771	286
Number of female calves retained as replacement heifers	−0.090	0.124	292
Interest in continuing the use or introducing extended lactation	−0.630	>0.001 **	274
Feasibility of extended lactation	−0.483	>0.001 **	284

* Correlation is significant at the level of 0.01. ** Correlation is significant at the level of 0.05.

**Table 4:** Herd type (breed) at 75% threshold.

	Fleckvieh (*n* = 131)	Holstein Friesian (*n* = 60)	Braunvieh (*n* = 33)	Vorder-Wälder (*n* = 8)	Other Breed (*n* = 9)	Mixed (50)	Total
Yes—extended lactation used	49	41	19	2	2	32	145
No—extended lactation is not used	82	19	14	6	7	18	146

**Table 5:** Interest in extended lactation and feasibility as marked on the 6-point Likert scale.

		Statistic	Std. Error
	*n*	284	
**Feasibility of extended lactation**	Mean	3.08	0.115
	Variance	2.495	
	Std. Deviation	1.579	
	Kurtosis	−0.816	0.288
	Skewness	0.418	0.145
	*n*	186	
**Interest in continuing the use or introducing extended lactation**	Mean	3.26	0.120
	Variance	2.839	
	Std. Deviation	1.685	
	Kurtosis	−1.154	0.293
	Skewness	0.284	0.147

**Table 6:** Correlation test using Pearson’s correlation against interest and feasibility.

Correlation Between:			
Interest in continuing the use/introducing extended lactation and:	Pearson correlation	*p*	*n*
Herd size	−0.035	0.561	273
Milk/cow/annum	−0.157	0.010 **	268
Milk production/farm/annum	−0.069	0.259	268
No. of female calves retained as replacement heifers	−0.010	0.865	272
**Feasibility of extended lactation and:**			
Number of cows in herd	−0.042	0.480	283
Milk/cow/annum	−0.135	0.024 *	278
Milk production/farm/annum	−0.067	0.267	278
No. of female calves retained as replacement heifers	−0.056	0.351	282

* Correlation is significant at the level of 0.01. ** Correlation is significant at the level of 0.05.

**Table 7:** Perceived potential of the strategy extended lactation.

Mean Scale Score	Farmers Currently Not Using Extended Lactation			Farmers Currently Using Extended Lactation			Sum
	Organic	Conv.	Sum	Organic	Conv.	Sum	
**1– 2.5** **(high to very high potential)**	14	3	17	64	28	92	109
	16.1%	6.5%	12.8%	64.6%	66.7%	65.2%	39.8%
**3–3.5** **(moderate potential)**	25	15	40	26	8	34	74
	28.7%	32.6%	30.1%	26.3%	19.0%	24.1%	27.0%
**4– 5.5** **(low to very low potential)**	33	22	55	9	6	15	70
	37.9%	47.8%	41.4%	9.1%	14.3%	10.6%	25.5%
**6** **(no potential)**	15	6	21	0	0	0	21
	17.2%	13.0%	15.8%				7.7%
**Sum**	**87**	**46**	**133**	**99**	**42**	**141**	**274**
							**100%**

**Table 8:** Four scenarios of extended lactation and the subsequent changes in calvings.

	Standard *	Scenario 1 3-Month Extension, Extending Productive Life	Scenario 2 6-Month Extension, Extending Productive Life	Scenario 3 3-Month Extension, Not Extending Productive Life	Scenario 4 6-Month Extension, Not Extending Productive Life
**Number of lactations**	4.1	4.1	4.1	3.37	2.86
**Lifespan in years**	6.6	7.61	8.62	6.6	6.6
**Calves/cow/annum of lifetime**	0.62	0.62	0.62	0.51	0.43
**Calves/cow/annum of productive life**	0.84	0.72	0.61	0.72	0.61
**Calving interval**	416	506	596	506	596
**Number of calves born**	269,194	249,427	231,137	249,427	231,137
**Annual reduction in calves born**	0%	−7.34%	−14.14%	−7.34%	−14.14%

* Based on LKV [31].

## Data Availability

Due to privacy restrictions the research data supporting the reported results cannot be shared publicly. The data are stored under current data storage regulations by the University of Hohenheim.
